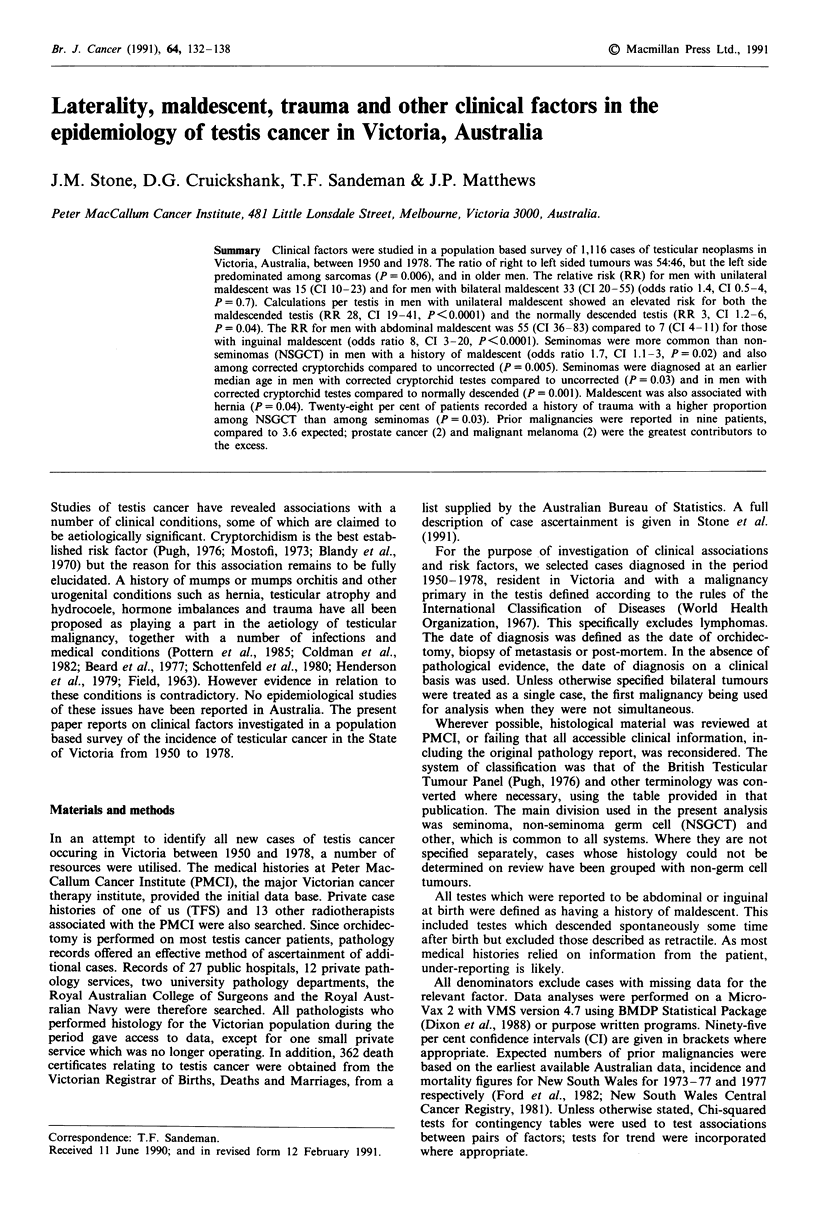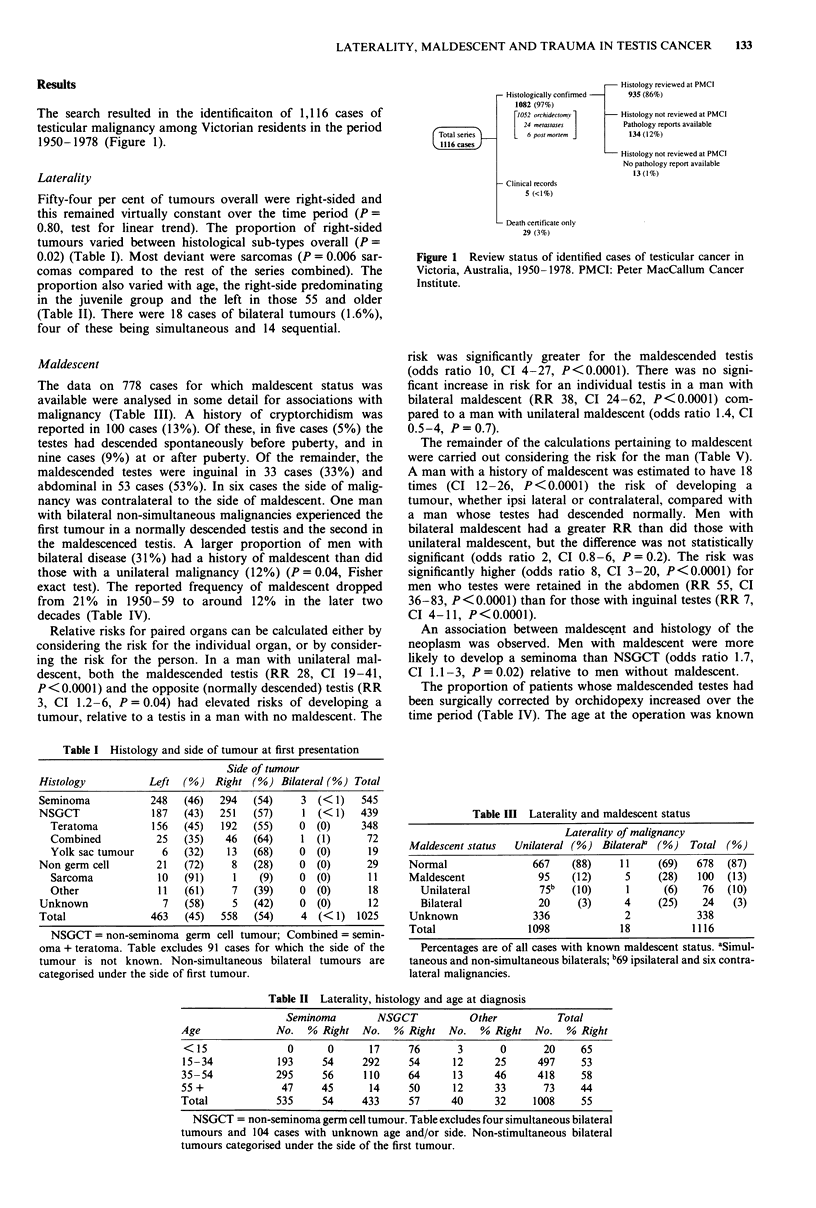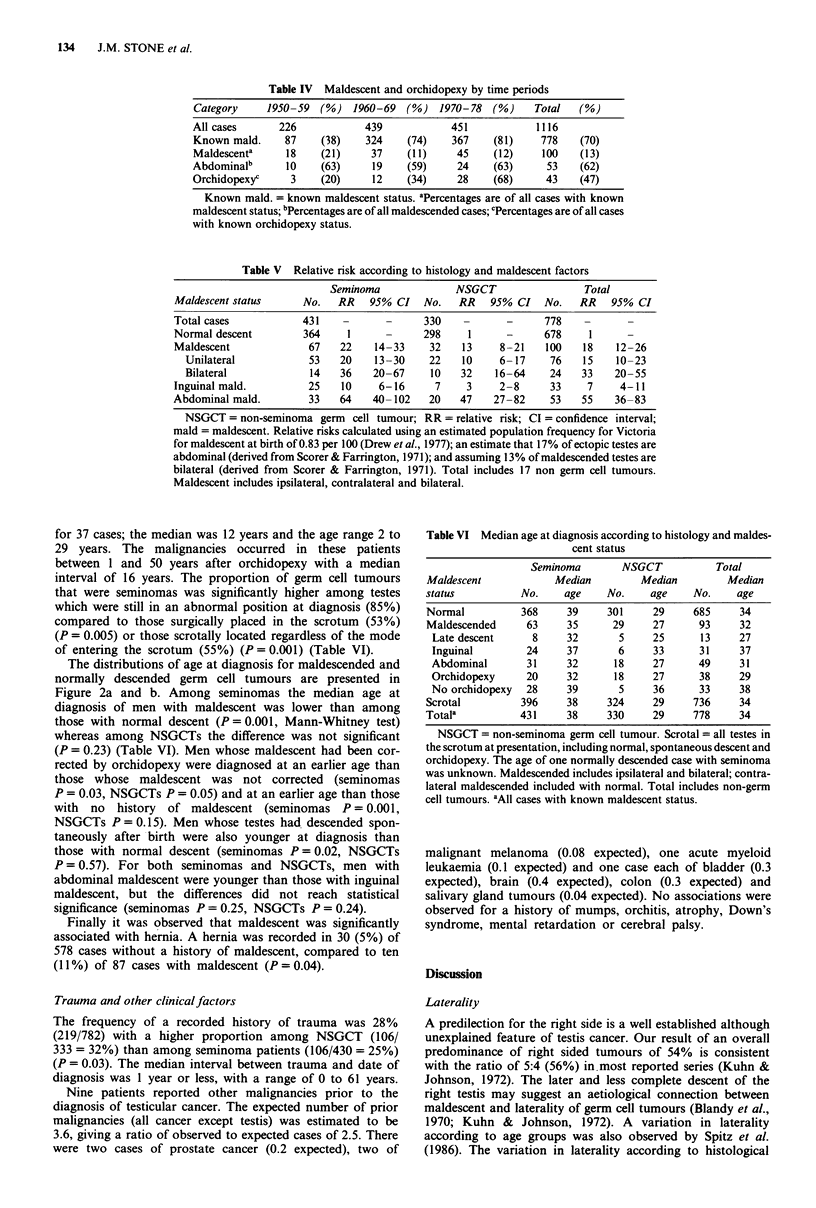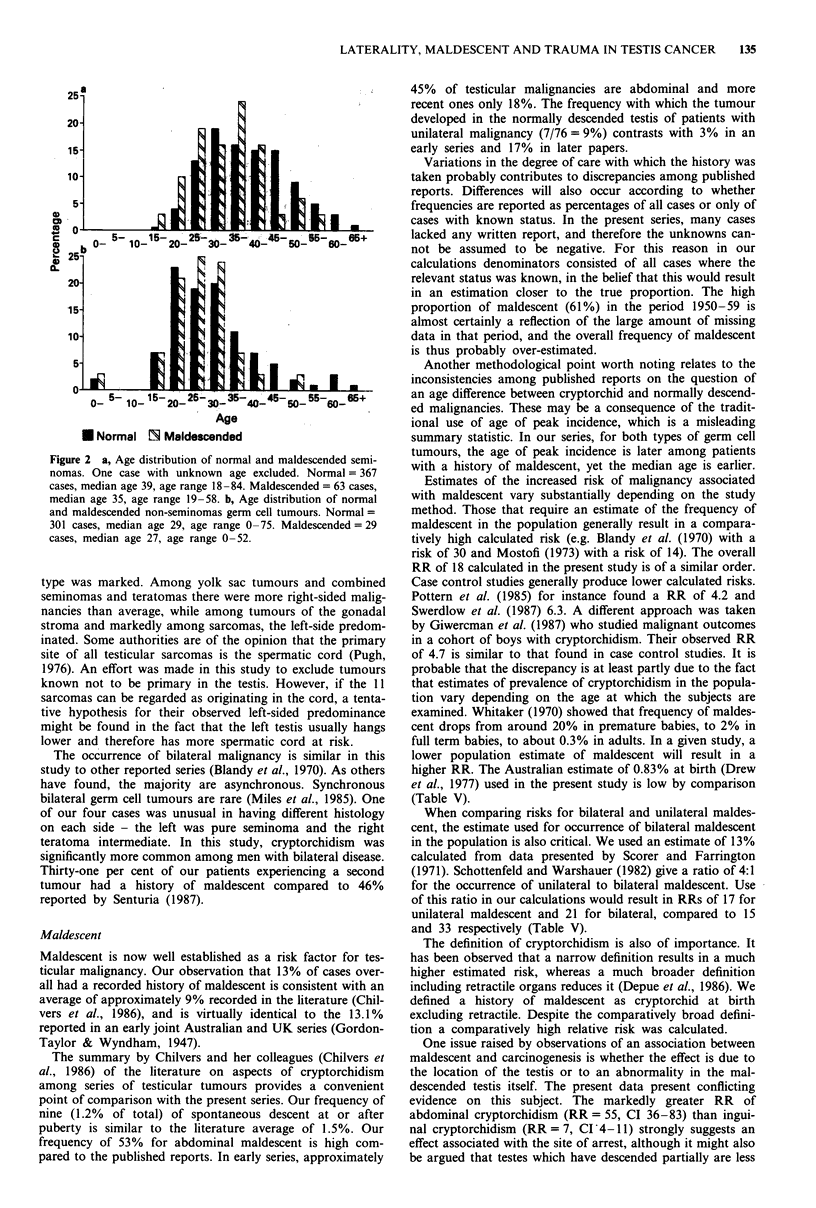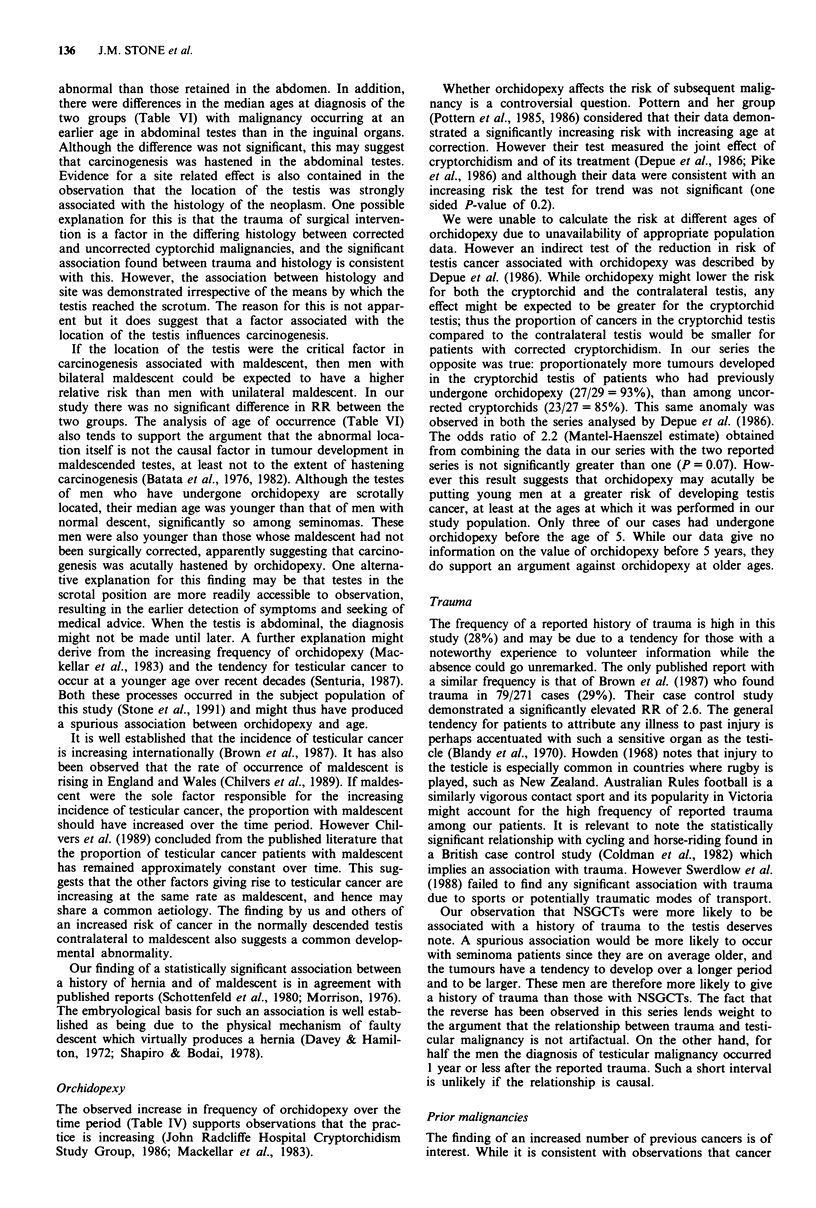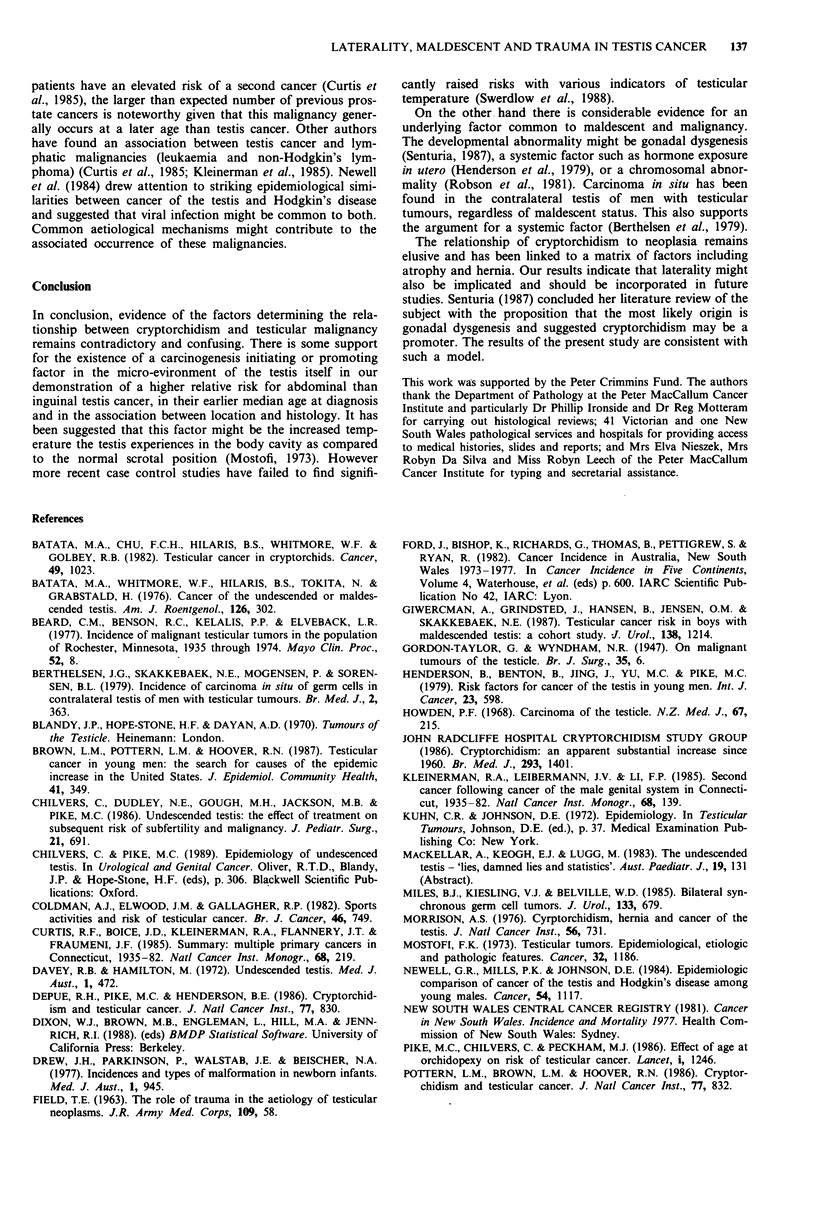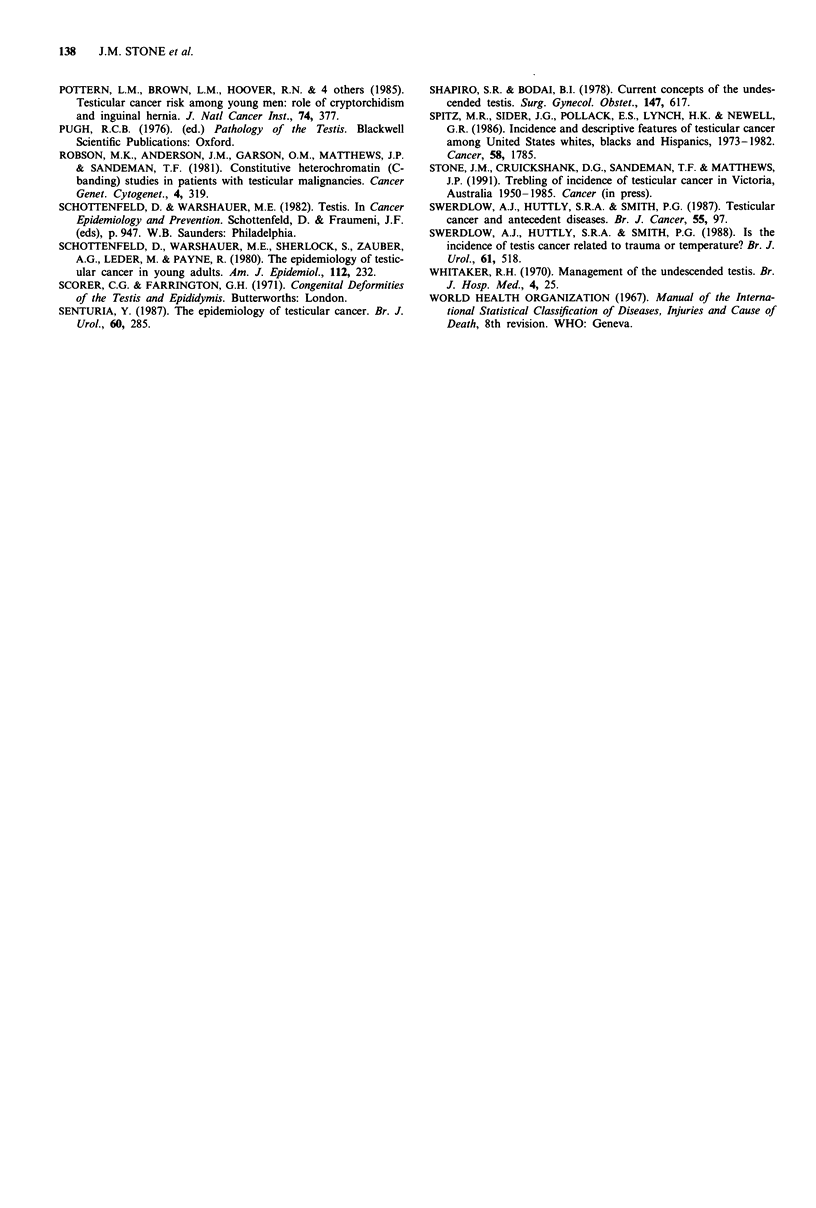# Laterality, maldescent, trauma and other clinical factors in the epidemiology of testis cancer in Victoria, Australia.

**DOI:** 10.1038/bjc.1991.256

**Published:** 1991-07

**Authors:** J. M. Stone, D. G. Cruickshank, T. F. Sandeman, J. P. Matthews

**Affiliations:** Peter MacCallum Cancer Institute, Melbourne, Victoria, Australia.

## Abstract

Clinical factors were studied in a population based survey of 1,116 cases of testicular neoplasms in Victoria, Australia, between 1950 and 1978. The ratio of right to left sided tumours was 54:46, but the left side predominated among sarcomas (P = 0.006), and in older men. The relative risk (RR) for men with unilateral maldescent was 15 (CI 10-23) and for men with bilateral maldescent 33 (CI 20-55) (odds ratio 1.4, CI 0.5-4, P = 0.7). Calculations per testis in men with unilateral maldescent showed an elevated risk for both the maldescended testis (RR 28, CI 19-41, P less than 0.0001) and the normally descended testis (RR 3, CI 1.2-6, P = 0.04). The RR for men with abdominal maldescent was 55 (CI 36-83) compared to 7 (CI 4-11) for those with inguinal maldescent (odds ratio 8, CI 3-20, P less than 0.0001). Seminomas were more common than nonseminomas (NSGCT) in men with a history of maldescent (odds ratio 1.7, CI 1.1-3, P = 0.02) and also among corrected cryptorchids compared to uncorrected (P = 0.005). Seminomas were diagnosed at an earlier median age in men with corrected cryptorchid testes compared to uncorrected (P = 0.03) and in men with corrected cryptorchid testes compared to normally descended (P = 0.001). Maldescent was also associated with hernia (P = 0.04). Twenty-eight per cent of patients recorded a history of trauma with a higher proportion among NSGCT than among seminomas (P = 0.03). Prior malignancies were reported in nine patients, compared to 3.6 expected; prostate cancer (2) and malignant melanoma (2) were the greatest contributors to the excess.


*Br. J. Cancer* (1991), **64**, 132–138 © Macmillan Press Ltd., 1991

### Laterality, maldescent, trauma and other clinical factors in the epidemiology of testis cancer in Victoria, Australia

J.M. Stone, D.G. Cruickshank, T.F. Sandeman & J.P. Matthews

*Peter MacCallum Cancer Institute, 481 Little Lonsdale Street, Melbourne, Victoria 3000, Australia.*

**Summary** Clinical factors were studied in a population based survey of 1,116 cases of testicular neoplasms in Victoria, Australia, between 1950 and 1978. The ratio of right to left sided tumours was 54:46, but the left side predominated among sarcomas ($P = 0.006$), and in older men. The relative risk (RR) for men with unilateral maldescent was 15 (CI 10–23) and for men with bilateral maldescent 33 (CI 20–55) (odds ratio 1.4, CI 0.5–4, [illegible] $P = 0.04$). The RR for men with abdominal maldescent was 55 (CI 36–83) compared to 7 (CI 4–11) for those with inguinal maldescent (odds ratio 8, CI 3–20, $P < 0.0001$). Seminomas were more common than non-seminomas (NSGCT) in men with a history of maldescent (odds ratio 1.7, CI 1.1–3, $P = 0.02$) and also among corrected cryptorchids compared to uncorrected ($P = 0.005$). Seminomas were diagnosed at an earlier median age in men with corrected cryptorchid testes compared to uncorrected ($P = 0.03$) and in men with corrected cryptorchid testes compared to normally descended ($P = 0.001$). Maldescent was also associated with hernia ($P = 0.04$). Twenty-eight per cent of patients recorded a history of trauma with a higher proportion among NSGCT than among seminomas ($P = 0.03$). Prior malignancies were reported in nine patients, compared to 3.6 expected; prostate cancer (2) and malignant melanoma (2) were the greatest contributors to the excess.

### Results

The search resulted in the identificaiton of 1,116 cases of testicular malignancy among Victorian residents in the period 1950–1978 (Figure 1).

**Figure 1** Review status of identified cases of testicular cancer in Victoria, Australia, 1950–1978. PMCI: Peter MacCallum Cancer Institute.

### *Laterality*

Fifty-four per cent of tumours overall were right-sided and this remained virtually constant over the time period ($P = 0.80$, test for linear trend). The proportion of right-sided tumours varied between histological sub-types overall ($P = 0.02$) (Table I). Most deviant were sarcomas ($P = 0.006$ sarcomas compared to the rest of the series combined). The proportion also varied with age, the right-side predominating in the juvenile group and the left in those 55 and older (Table II). There were 18 cases of bilateral tumours (1.6%), four of these being simultaneous and 14 sequential.

### *Maldescent*

The data on 778 cases for which maldescent status was available were analysed in some detail for associations with malignancy (Table III). A history of cryptorchidism was reported in 100 cases (13%). Of these, in five cases (5%) the testes had descended spontaneously before puberty, and in nine cases (9%) at or after puberty. Of the remainder, the maldescended testes were inguinal in 33 cases (33%) and abdominal in 53 cases (53%). In six cases the side of malignancy was contralateral to the side of maldescent. One man with bilateral non-simultaneous malignancies experienced the first tumour in a normally descended testis and the second in the maldescenced testis. A larger proportion of men with bilateral disease (31%) had a history of maldescent than did those with a unilateral malignancy (12%) ($P = 0.04$, Fisher exact test). The reported frequency of maldescent dropped from 21% in 1950–59 to around 12% in the later two decades (Table IV).

Relative risks for paired organs can be calculated either by considering the risk for the individual organ, or by considering the risk for the person. In a man with unilateral maldescent, both the maldescended testis (RR 28, CI 19–41, $P < 0.0001$) and the opposite (normally descended) testis (RR 3, CI 1.2–6, $P = 0.04$) had elevated risks of developing a tumour, relative to a testis in a man with no maldescent. The risk was significantly greater for the maldescended testis (odds ratio 10, CI 4–27, $P < 0.0001$). There was no significant increase in risk for an individual testis in a man with bilateral maldescent (RR 38, CI 24–62, $P < 0.0001$) compared to a man with unilateral maldescent (odds ratio 1.4, CI 0.5–4, $P = 0.7$).

The remainder of the calculations pertaining to maldescent were carried out considering the risk for the man (Table V). A man with a history of maldescent was estimated to have 18 times (CI 12–26, $P < 0.0001$) the risk of developing a tumour, whether ipsi lateral or contralateral, compared with a man whose testes had descended normally. Men with bilateral maldescent had a greater RR than did those with unilateral maldescent, but the difference was not statistically significant (odds ratio 2, CI 0.8–6, $P = 0.2$). The risk was significantly higher (odds ratio 8, CI 3–20, $P < 0.0001$) for men who testes were retained in the abdomen (RR 55, CI 36–83, $P < 0.0001$) than for those with inguinal testes (RR 7, CI 4–11, $P < 0.0001$).

An association between maldescent and histology of the neoplasm was observed. Men with maldescent were more likely to develop a seminoma than NSGCT (odds ratio 1.7, CI 1.1–3, $P = 0.02$) relative to men without maldescent.

The proportion of patients whose maldescended testes had been surgically corrected by orchidopexy increased over the time period (Table IV). The age at the operation was known

**Table I** Histology and side of tumour at first presentation

| *Histology* | *Left* | *(%)* | *Right* | *(%)* | *Bilateral* | *(%)* | *Total* |
|---|---|---|---|---|---|---|---|
| | *Side of tumour* | | | | | | |
| Seminoma | 248 | (46) | 294 | (54) | 3 | (<1) | 545 |
| NSGCT | 187 | (43) | 251 | (57) | 1 | (<1) | 439 |
| Teratoma | 156 | (45) | 192 | (55) | 0 | (0) | 348 |
| Combined | 25 | (35) | 46 | (64) | 1 | (1) | 72 |
| Yolk sac tumour | 6 | (32) | 13 | (68) | 0 | (0) | 19 |
| Non germ cell | 21 | (72) | 8 | (28) | 0 | (0) | 29 |
| Sarcoma | 10 | (91) | 1 | (9) | 0 | (0) | 11 |
| Other | 11 | (61) | 7 | (39) | 0 | (0) | 18 |
| Unknown | 7 | (58) | 5 | (42) | 0 | (0) | 12 |
| Total | 463 | (45) | 558 | (54) | 4 | (<1) | 1025 |

NSGCT = non-seminoma germ cell tumour; Combined = seminoma + teratoma. Table excludes 91 cases for which the side of the tumour is not known. Non-simultaneous bilateral tumours are categorised under the side of first tumour.

**Table III** Laterality and maldescent status

| *Maldescent status* | *Unilateral* | *(%)* | *Bilateral*[a] | *(%)* | *Total* | *(%)* |
|---|---|---|---|---|---|---|
| | *Laterality of malignancy* | | | | | |
| Normal | 667 | (88) | 11 | (69) | 678 | (87) |
| Maldescent | 95 | (12) | 5 | (28) | 100 | (13) |
| Unilateral | 75[b] | (10) | 1 | (6) | 76 | (10) |
| Bilateral | 20 | (3) | 4 | (25) | 24 | (3) |
| Unknown | 336 | | 2 | | 338 | |
| Total | 1098 | | 18 | | 1116 | |

Percentages are of all cases with known maldescent status. [a]Simultaneous and non-simultaneous bilaterals; [b]69 ipsilateral and six contralateral malignancies.

**Table II** Laterality, histology and age at diagnosis

| *Age* | *Seminoma No.* | *% Right* | *NSGCT No.* | *% Right* | *Other No.* | *% Right* | *Total No.* | *% Right* |
|---|---|---|---|---|---|---|---|---|
| <15 | 0 | 0 | 17 | 76 | 3 | 0 | 20 | 65 |
| 15–34 | 193 | 54 | 292 | 54 | 12 | 25 | 497 | 53 |
| 35–54 | 295 | 56 | 110 | 64 | 13 | 46 | 418 | 58 |
| 55 + | 47 | 45 | 14 | 50 | 12 | 33 | 73 | 44 |
| Total | 535 | 54 | 433 | 57 | 40 | 32 | 1008 | 55 |

NSGCT = non-seminoma germ cell tumour. Table excludes four simultaneous bilateral tumours and 104 cases with unknown age and/or side. Non-stimultaneous bilateral tumours categorised under the side of the first tumour.

Table IV Maldescent and orchidopexy by time periods

| Category | 1950-59 | (%) | 1960-69 | (%) | 1970-78 | (%) | Total | (%) |
|---|---|---|---|---|---|---|---|---|
| All cases | 226 | | 439 | | 451 | | 1116 | |
| Known mald. | 87 | (38) | 324 | (74) | 367 | (81) | 778 | (70) |
| Maldescent[a] | 18 | (21) | 37 | (11) | 45 | (12) | 100 | (13) |
| Abdominal[b] | 10 | (63) | 19 | (59) | 24 | (63) | 53 | (62) |
| Orchidopexy[c] | 3 | (20) | 12 | (34) | 28 | (68) | 43 | (47) |

Known mald. = known maldescent status. [a]Percentages are of all cases with known maldescent status; [b]Percentages are of all maldescended cases; [c]Percentages are of all cases with known orchidopexy status.

Table V Relative risk according to histology and maldescent factors

| Maldescent status | Seminoma No. | Seminoma RR | Seminoma 95% CI | NSGCT No. | NSGCT RR | NSGCT 95% CI | Total No. | Total RR | Total 95% CI |
|---|---|---|---|---|---|---|---|---|---|
| Total cases | 431 | – | – | 330 | – | – | 778 | – | – |
| Normal descent | 364 | 1 | – | 298 | 1 | – | 678 | 1 | – |
| Maldescent | 67 | 22 | 14–33 | 32 | 13 | 8–21 | 100 | 18 | 12–26 |
| Unilateral | 53 | 20 | 13–30 | 22 | 10 | 6–17 | 76 | 15 | 10–23 |
| Bilateral | 14 | 36 | 20–67 | 10 | 32 | 16–64 | 24 | 33 | 20–55 |
| Inguinal mald. | 25 | 10 | 6–16 | 7 | 3 | 2–8 | 33 | 7 | 4–11 |
| Abdominal mald. | 33 | 64 | 40–102 | 20 | 47 | 27–82 | 53 | 55 | 36–83 |

NSGCT = non-seminoma germ cell tumour; RR = relative risk; CI = confidence interval; mald = maldescent. Relative risks calculated using an estimated population frequency for Victoria for maldescent at birth of 0.83 per 100 (Drew et al., 1977); an estimate that 17% of ectopic testes are abdominal (derived from Scorer & Farrington, 1971); and assuming 13% of maldescended testes are bilateral (derived from Scorer & Farrington, 1971). Total includes 17 non germ cell tumours. Maldescent includes ipsilateral, contralateral and bilateral.

for 37 cases; the median was 12 years and the age range 2 to 29 years. The malignancies occurred in these patients between 1 and 50 years after orchidopexy with a median interval of 16 years. The proportion of germ cell tumours that were seminomas was significantly higher among testes which were still in an abnormal position at diagnosis (85%) compared to those surgically placed in the scrotum (53%) ($P = 0.005$) or those scrotally located regardless of the mode of entering the scrotum (55%) ($P = 0.001$) (Table VI).

The distributions of age at diagnosis for maldescended and normally descended germ cell tumours are presented in Figure 2a and b. Among seminomas the median age at diagnosis of men with maldescent was lower than among those with normal descent ($P = 0.001$, Mann-Whitney test) whereas among NSGCTs the difference was not significant ($P = 0.23$) (Table VI). Men whose maldescent had been corrected by orchidopexy were diagnosed at an earlier age than those whose maldescent was not corrected (seminomas $P = 0.03$, NSGCTs $P = 0.05$) and at an earlier age than those with no history of maldescent (seminomas $P = 0.001$, NSGCTs $P = 0.15$). Men whose testes had descended spontaneously after birth were also younger at diagnosis than those with normal descent (seminomas $P = 0.02$, NSGCTs $P = 0.57$). For both seminomas and NSGCTs, men with abdominal maldescent were younger than those with inguinal maldescent, but the differences did not reach statistical significance (seminomas $P = 0.25$, NSGCTs $P = 0.24$).

Finally it was observed that maldescent was significantly associated with hernia. A hernia was recorded in 30 (5%) of 578 cases without a history of maldescent, compared to ten (11%) of 87 cases with maldescent ($P = 0.04$).

### *Trauma and other clinical factors*

The frequency of a recorded history of trauma was 28% (219/782) with a higher proportion among NSGCT (106/333 = 32%) than among seminoma patients (106/430 = 25%) ($P = 0.03$). The median interval between trauma and date of diagnosis was 1 year or less, with a range of 0 to 61 years.

Nine patients reported other malignancies prior to the diagnosis of testicular cancer. The expected number of prior malignancies (all cancer except testis) was estimated to be 3.6, giving a ratio of observed to expected cases of 2.5. There were two cases of prostate cancer (0.2 expected), two of malignant melanoma (0.08 expected), one acute myeloid leukaemia (0.1 expected) and one case each of bladder (0.3 expected), brain (0.4 expected), colon (0.3 expected) and salivary gland tumours (0.04 expected). No associations were observed for a history of mumps, orchitis, atrophy, Down's syndrome, mental retardation or cerebral palsy.

Table VI Median age at diagnosis according to histology and maldescent status

| Maldescent status | Seminoma No. | Seminoma Median age | NSGCT No. | NSGCT Median age | Total No. | Total Median age |
|---|---|---|---|---|---|---|
| Normal | 368 | 39 | 301 | 29 | 685 | 34 |
| Maldescended | 63 | 35 | 29 | 27 | 93 | 32 |
| Late descent | 8 | 32 | 5 | 25 | 13 | 27 |
| Inguinal | 24 | 37 | 6 | 33 | 31 | 37 |
| Abdominal | 31 | 32 | 18 | 27 | 49 | 31 |
| Orchidopexy | 20 | 32 | 18 | 27 | 38 | 29 |
| No orchidopexy | 28 | 39 | 5 | 36 | 33 | 38 |
| Scrotal | 396 | 38 | 324 | 29 | 736 | 34 |
| Total[a] | 431 | 38 | 330 | 29 | 778 | 34 |

NSGCT = non-seminoma germ cell tumour. Scrotal = all testes in the scrotum at presentation, including normal, spontaneous descent and orchidopexy. The age of one normally descended case with seminoma was unknown. Maldescended includes ipsilateral and bilateral; contralateral maldescended included with normal. Total includes non-germ cell tumours. [a]All cases with known maldescent status.

### Discussion

### *Laterality*

A predilection for the right side is a well established although unexplained feature of testis cancer. Our result of an overall predominance of right sided tumours of 54% is consistent with the ratio of 5:4 (56%) in most reported series (Kuhn & Johnson, 1972). The later and less complete descent of the right testis may suggest an aetiological connection between maldescent and laterality of germ cell tumours (Blandy et al., 1970; Kuhn & Johnson, 1972). A variation in laterality according to age groups was also observed by Spitz et al. (1986). The variation in laterality according to histological

**Figure 2** **a,** Age distribution of normal and maldescended seminomas. One case with unknown age excluded. Normal = 367 cases, median age 39, age range 18–84. Maldescended = 63 cases, median age 35, age range 19–58. **b,** Age distribution of normal and maldescended non-seminomas germ cell tumours. Normal = 301 cases, median age 29, age range 0–75. Maldescended = 29 cases, median age 27, age range 0–52.

type was marked. Among yolk sac tumours and combined seminomas and teratomas there were more right-sided malignancies than average, while among tumours of the gonadal stroma and markedly among sarcomas, the left-side predominated. Some authorities are of the opinion that the primary site of all testicular sarcomas is the spermatic cord (Pugh, 1976). An effort was made in this study to exclude tumours known not to be primary in the testis. However, if the 11 sarcomas can be regarded as originating in the cord, a tentative hypothesis for their observed left-sided predominance might be found in the fact that the left testis usually hangs lower and therefore has more spermatic cord at risk.

The occurrence of bilateral malignancy is similar in this study to other reported series (Blandy *et al.*, 1970). As others have found, the majority are asynchronous. Synchronous bilateral germ cell tumours are rare (Miles *et al.*, 1985). One of our four cases was unusual in having different histology on each side – the left was pure seminoma and the right teratoma intermediate. In this study, cryptorchidism was significantly more common among men with bilateral disease. Thirty-one per cent of our patients experiencing a second tumour had a history of maldescent compared to 46% reported by Senturia (1987).

*Maldescent*

Maldescent is now well established as a risk factor for testicular malignancy. Our observation that 13% of cases overall had a recorded history of maldescent is consistent with an average of approximately 9% recorded in the literature (Chilvers *et al.*, 1986), and is virtually identical to the 13.1% reported in an early joint Australian and UK series (Gordon-Taylor & Wyndham, 1947).

The summary by Chilvers and her colleagues (Chilvers *et al.*, 1986) of the literature on aspects of cryptorchidism among series of testicular tumours provides a convenient point of comparison with the present series. Our frequency of nine (1.2% of total) of spontaneous descent at or after puberty is similar to the literature average of 1.5%. Our frequency of 53% for abdominal maldescent is high compared to the published reports. In early series, approximately 45% of testicular malignancies are abdominal and more recent ones only 18%. The frequency with which the tumour developed in the normally descended testis of patients with unilateral malignancy (7/76 = 9%) contrasts with 3% in an early series and 17% in later papers.

Variations in the degree of care with which the history was taken probably contributes to discrepancies among published reports. Differences will also occur according to whether frequencies are reported as percentages of all cases or only of cases with known status. In the present series, many cases lacked any written report, and therefore the unknowns cannot be assumed to be negative. For this reason in our calculations denominators consisted of all cases where the relevant status was known, in the belief that this would result in an estimation closer to the true proportion. The high proportion of maldescent (61%) in the period 1950–59 is almost certainly a reflection of the large amount of missing data in that period, and the overall frequency of maldescent is thus probably over-estimated.

Another methodological point worth noting relates to the inconsistencies among published reports on the question of an age difference between cryptorchid and normally descended malignancies. These may be a consequence of the traditional use of age of peak incidence, which is a misleading summary statistic. In our series, for both types of germ cell tumours, the age of peak incidence is later among patients with a history of maldescent, yet the median age is earlier.

Estimates of the increased risk of malignancy associated with maldescent vary substantially depending on the study method. Those that require an estimate of the frequency of maldescent in the population generally result in a comparatively high calculated risk (e.g. Blandy *et al.* (1970) with a risk of 30 and Mostofi (1973) with a risk of 14). The overall RR of 18 calculated in the present study is of a similar order. Case control studies generally produce lower calculated risks. Pottern *et al.* (1985) for instance found a RR of 4.2 and Swerdlow *et al.* (1987) 6.3. A different approach was taken by Giwercman *et al.* (1987) who studied malignant outcomes in a cohort of boys with cryptorchidism. Their observed RR of 4.7 is similar to that found in case control studies. It is probable that the discrepancy is at least partly due to the fact that estimates of prevalence of cryptorchidism in the population vary depending on the age at which the subjects are examined. Whitaker (1970) showed that frequency of maldescent drops from around 20% in premature babies, to 2% in full term babies, to about 0.3% in adults. In a given study, a lower population estimate of maldescent will result in a higher RR. The Australian estimate of 0.83% at birth (Drew *et al.*, 1977) used in the present study is low by comparison (Table V).

When comparing risks for bilateral and unilateral maldescent, the estimate used for occurrence of bilateral maldescent in the population is also critical. We used an estimate of 13% calculated from data presented by Scorer and Farrington (1971). Schottenfeld and Warshauer (1982) give a ratio of 4:1 for the occurrence of unilateral to bilateral maldescent. Use of this ratio in our calculations would result in RRs of 17 for unilateral maldescent and 21 for bilateral, compared to 15 and 33 respectively (Table V).

The definition of cryptorchidism is also of importance. It has been observed that a narrow definition results in a much higher estimated risk, whereas a much broader definition including retractile organs reduces it (Depue *et al.*, 1986). We defined a history of maldescent as cryptorchid at birth excluding retractile. Despite the comparatively broad definition a comparatively high relative risk was calculated.

One issue raised by observations of an association between maldescent and carcinogenesis is whether the effect is due to the location of the testis or to an abnormality in the maldescended testis itself. The present data present conflicting evidence on this subject. The markedly greater RR of abdominal cryptorchidism (RR = 55, CI 36–83) than inguinal cryptorchidism (RR = 7, CI 4–11) strongly suggests an effect associated with the site of arrest, although it might also be argued that testes which have descended partially are less abnormal than those retained in the abdomen. In addition, there were differences in the median ages at diagnosis of the two groups (Table VI) with malignancy occurring at an earlier age in abdominal testes than in the inguinal organs. Although the difference was not significant, this may suggest that carcinogenesis was hastened in the abdominal testes. Evidence for a site related effect is also contained in the observation that the location of the testis was strongly associated with the histology of the neoplasm. One possible explanation for this is that the trauma of surgical intervention is a factor in the differing histology between corrected and uncorrected cyptorchid malignancies, and the significant association found between trauma and histology is consistent with this. However, the association between histology and site was demonstrated irrespective of the means by which the testis reached the scrotum. The reason for this is not apparent but it does suggest that a factor associated with the location of the testis influences carcinogenesis.

If the location of the testis were the critical factor in carcinogenesis associated with maldescent, then men with bilateral maldescent could be expected to have a higher relative risk than men with unilateral maldescent. In our study there was no significant difference in RR between the two groups. The analysis of age of occurrence (Table VI) also tends to support the argument that the abnormal location itself is not the causal factor in tumour development in maldescended testes, at least not to the extent of hastening carcinogenesis (Batata *et al.*, 1976, 1982). Although the testes of men who have undergone orchidopexy are scrotally located, their median age was younger than that of men with normal descent, significantly so among seminomas. These men were also younger than those whose maldescent had not been surgically corrected, apparently suggesting that carcinogenesis was acutally hastened by orchidopexy. One alternative explanation for this finding may be that testes in the scrotal position are more readily accessible to observation, resulting in the earlier detection of symptoms and seeking of medical advice. When the testis is abdominal, the diagnosis might not be made until later. A further explanation might derive from the increasing frequency of orchidopexy (Mackellar *et al.*, 1983) and the tendency for testicular cancer to occur at a younger age over recent decades (Senturia, 1987). Both these processes occurred in the subject population of this study (Stone *et al.*, 1991) and might thus have produced a spurious association between orchidopexy and age.

It is well established that the incidence of testicular cancer is increasing internationally (Brown *et al.*, 1987). It has also been observed that the rate of occurrence of maldescent is rising in England and Wales (Chilvers *et al.*, 1989). If maldescent were the sole factor responsible for the increasing incidence of testicular cancer, the proportion with maldescent should have increased over the time period. However Chilvers *et al.* (1989) concluded from the published literature that the proportion of testicular cancer patients with maldescent has remained approximately constant over time. This suggests that the other factors giving rise to testicular cancer are increasing at the same rate as maldescent, and hence may share a common aetiology. The finding by us and others of an increased risk of cancer in the normally descended testis contralateral to maldescent also suggests a common developmental abnormality.

Our finding of a statistically significant association between a history of hernia and of maldescent is in agreement with published reports (Schottenfeld *et al.*, 1980; Morrison, 1976). The embryological basis for such an association is well established as being due to the physical mechanism of faulty descent which virtually produces a hernia (Davey & Hamilton, 1972; Shapiro & Bodai, 1978).

### *Orchidopexy*

The observed increase in frequency of orchidopexy over the time period (Table IV) supports observations that the practice is increasing (John Radcliffe Hospital Cryptorchidism Study Group, 1986; Mackellar *et al.*, 1983).

Whether orchidopexy affects the risk of subsequent malignancy is a controversial question. Pottern and her group (Pottern *et al.*, 1985, 1986) considered that their data demonstrated a significantly increasing risk with increasing age at correction. However their test measured the joint effect of cryptorchidism and of its treatment (Depue *et al.*, 1986; Pike *et al.*, 1986) and although their data were consistent with an increasing risk the test for trend was not significant (one sided *P*-value of 0.2).

We were unable to calculate the risk at different ages of orchidopexy due to unavailability of appropriate population data. However an indirect test of the reduction in risk of testis cancer associated with orchidopexy was described by Depue *et al.* (1986). While orchidopexy might lower the risk for both the cryptorchid and the contralateral testis, any effect might be expected to be greater for the cryptorchid testis; thus the proportion of cancers in the cryptorchid testis compared to the contralateral testis would be smaller for patients with corrected cryptorchidism. In our series the opposite was true: proportionately more tumours developed in the cryptorchid testis of patients who had previously undergone orchidopexy ($27/29 = 93\%$), than among uncorrected cryptorchids ($23/27 = 85\%$). This same anomaly was observed in both the series analysed by Depue *et al.* (1986). The odds ratio of 2.2 (Mantel-Haenszel estimate) obtained from combining the data in our series with the two reported series is not significantly greater than one ($P = 0.07$). However this result suggests that orchidopexy may acutally be putting young men at a greater risk of developing testis cancer, at least at the ages at which it was performed in our study population. Only three of our cases had undergone orchidopexy before the age of 5. While our data give no information on the value of orchidopexy before 5 years, they do support an argument against orchidopexy at older ages.

### *Trauma*

The frequency of a reported history of trauma is high in this study (28%) and may be due to a tendency for those with a noteworthy experience to volunteer information while the absence could go unremarked. The only published report with a similar frequency is that of Brown *et al.* (1987) who found trauma in 79/271 cases (29%). Their case control study demonstrated a significantly elevated RR of 2.6. The general tendency for patients to attribute any illness to past injury is perhaps accentuated with such a sensitive organ as the testicle (Blandy *et al.*, 1970). Howden (1968) notes that injury to the testicle is especially common in countries where rugby is played, such as New Zealand. Australian Rules football is a similarly vigorous contact sport and its popularity in Victoria might account for the high frequency of reported trauma among our patients. It is relevant to note the statistically significant relationship with cycling and horse-riding found in a British case control study (Coldman *et al.*, 1982) which implies an association with trauma. However Swerdlow *et al.* (1988) failed to find any significant association with trauma due to sports or potentially traumatic modes of transport.

Our observation that NSGCTs were more likely to be associated with a history of trauma to the testis deserves note. A spurious association would be more likely to occur with seminoma patients since they are on average older, and the tumours have a tendency to develop over a longer period and to be larger. These men are therefore more likely to give a history of trauma than those with NSGCTs. The fact that the reverse has been observed in this series lends weight to the argument that the relationship between trauma and testicular malignancy is not artifactual. On the other hand, for half the men the diagnosis of testicular malignancy occurred 1 year or less after the reported trauma. Such a short interval is unlikely if the relationship is causal.

### *Prior malignancies*

The finding of an increased number of previous cancers is of interest. While it is consistent with observations that cancer patients have an elevated risk of a second cancer (Curtis *et al.*, 1985), the larger than expected number of previous prostate cancers is noteworthy given that this malignancy generally occurs at a later age than testis cancer. Other authors have found an association between testis cancer and lymphatic malignancies (leukaemia and non-Hodgkin's lymphoma) (Curtis *et al.*, 1985; Kleinerman *et al.*, 1985). Newell *et al.* (1984) drew attention to striking epidemiological similarities between cancer of the testis and Hodgkin's disease and suggested that viral infection might be common to both. Common aetiological mechanisms might contribute to the associated occurrence of these malignancies.

### Conclusion

In conclusion, evidence of the factors determining the relationship between cryptorchidism and testicular malignancy remains contradictory and confusing. There is some support for the existence of a carcinogenesis initiating or promoting factor in the micro-evironment of the testis itself in our demonstration of a higher relative risk for abdominal than inguinal testis cancer, in their earlier median age at diagnosis and in the association between location and histology. It has been suggested that this factor might be the increased temperature the testis experiences in the body cavity as compared to the normal scrotal position (Mostofi, 1973). However more recent case control studies have failed to find significantly raised risks with various indicators of testicular temperature (Swerdlow *et al.*, 1988).

On the other hand there is considerable evidence for an underlying factor common to maldescent and malignancy. The developmental abnormality might be gonadal dysgenesis (Senturia, 1987), a systemic factor such as hormone exposure *in utero* (Henderson *et al.*, 1979), or a chromosomal abnormality (Robson *et al.*, 1981). Carcinoma *in situ* has been found in the contralateral testis of men with testicular tumours, regardless of maldescent status. This also supports the argument for a systemic factor (Berthelsen *et al.*, 1979).

The relationship of cryptorchidism to neoplasia remains elusive and has been linked to a matrix of factors including atrophy and hernia. Our results indicate that laterality might also be implicated and should be incorporated in future studies. Senturia (1987) concluded her literature review of the subject with the proposition that the most likely origin is gonadal dysgenesis and suggested cryptorchidism may be a promoter. The results of the present study are consistent with such a model.

This work was supported by the Peter Crimmins Fund. The authors thank the Department of Pathology at the Peter MacCallum Cancer Institute and particularly Dr Phillip Ironside and Dr Reg Motteram for carrying out histological reviews; 41 Victorian and one New South Wales pathological services and hospitals for providing access to medical histories, slides and reports; and Mrs Elva Nieszek, Mrs Robyn Da Silva and Miss Robyn Leech of the Peter MacCallum Cancer Institute for typing and secretarial assistance.

### References

BATATA, M.A., CHU, F.C.H., HILARIS, B.S., WHITMORE, W.F. & GOLBEY, R.B. (1982). Testicular cancer in cryptorchids. *Cancer*, **49**, 1023.

BATATA, M.A., WHITMORE, W.F., HILARIS, B.S., TOKITA, N. & GRABSTALD, H. (1976). Cancer of the undescended or maldescended testis. *Am. J. Roentgenol.*, **126**, 302.

BEARD, C.M., BENSON, R.C., KELALIS, P.P. & ELVEBACK, L.R. (1977). Incidence of malignant testicular tumors in the population of Rochester, Minnesota, 1935 through 1974. *Mayo Clin. Proc.*, **52**, 8.

BERTHELSEN, J.G., SKAKKEBAEK, N.E., MOGENSEN, P. & SORENSEN, B.L. (1979). Incidence of carcinoma *in situ* of germ cells in contralateral testis of men with testicular tumours. *Br. Med. J.*, **2**, 363.

BLANDY, J.P., HOPE-STONE, H.F. & DAYAN, A.D. (1970). *Tumours of the Testicle*. Heinemann: London.

BROWN, L.M., POTTERN, L.M. & HOOVER, R.N. (1987). Testicular cancer in young men: the search for causes of the epidemic increase in the United States. *J. Epidemiol. Community Health*, **41**, 349.

CHILVERS, C., DUDLEY, N.E., GOUGH, M.H., JACKSON, M.B. & PIKE, M.C. (1986). Undescended testis: the effect of treatment on subsequent risk of subfertility and malignancy. *J. Pediatr. Surg.*, **21**, 691.

CHILVERS, C. & PIKE, M.C. (1989). Epidemiology of undescenced testis. In *Urological and Genital Cancer*. Oliver, R.T.D., Blandy, J.P. & Hope-Stone, H.F. (eds), p. 306. Blackwell Scientific Publications: Oxford.

COLDMAN, A.J., ELWOOD, J.M. & GALLAGHER, R.P. (1982). Sports activities and risk of testicular cancer. *Br. J. Cancer*, **46**, 749.

CURTIS, R.F., BOICE, J.D., KLEINERMAN, R.A., FLANNERY, J.T. & FRAUMENI, J.F. (1985). Summary: multiple primary cancers in Connecticut, 1935–82. *Natl Cancer Inst. Monogr.*, **68**, 219.

DAVEY, R.B. & HAMILTON, M. (1972). Undescended testis. *Med. J. Aust.*, **1**, 472.

DEPUE, R.H., PIKE, M.C. & HENDERSON, B.E. (1986). Cryptorchidism and testicular cancer. *J. Natl Cancer Inst.*, **77**, 830.

DIXON, W.J., BROWN, M.B., ENGLEMAN, L., HILL, M.A. & JENNRICH, R.I. (1988). (eds) *BMDP Statistical Software*. University of California Press: Berkeley.

DREW, J.H., PARKINSON, P., WALSTAB, J.E. & BEISCHER, N.A. (1977). Incidences and types of malformation in newborn infants. *Med. J. Aust.*, **1**, 945.

FIELD, T.E. (1963). The role of trauma in the aetiology of testicular neoplasms. *J.R. Army Med. Corps*, **109**, 58.

FORD, J., BISHOP, K., RICHARDS, G., THOMAS, B., PETTIGREW, S. & RYAN, R. (1982). Cancer Incidence in Australia, New South Wales 1973–1977. In *Cancer Incidence in Five Continents*, Volume 4, Waterhouse, *et al.* (eds) p. 600. IARC Scientific Publication No 42, IARC: Lyon.

GIWERCMAN, A., GRINDSTED, J., HANSEN, B., JENSEN, O.M. & SKAKKEBAEK, N.E. (1987). Testicular cancer risk in boys with maldescended testis: a cohort study. *J. Urol.*, **138**, 1214.

GORDON-TAYLOR, G. & WYNDHAM, N.R. (1947). On malignant tumours of the testicle. *Br. J. Surg.*, **35**, 6.

HENDERSON, B., BENTON, B., JING, J., YU, M.C. & PIKE, M.C. (1979). Risk factors for cancer of the testis in young men. *Int. J. Cancer*, **23**, 598.

HOWDEN, P.F. (1968). Carcinoma of the testicle. *N.Z. Med. J.*, **67**, 215.

JOHN RADCLIFFE HOSPITAL CRYPTORCHIDISM STUDY GROUP (1986). Cryptorchidism: an apparent substantial increase since 1960. *Br. Med. J.*, **293**, 1401.

KLEINERMAN, R.A., LEIBERMANN, J.V. & LI, F.P. (1985). Second cancer following cancer of the male genital system in Connecticut, 1935–82. *Natl Cancer Inst. Monogr.*, **68**, 139.

KUHN, C.R. & JOHNSON, D.E. (1972). Epidemiology. In *Testicular Tumours*, Johnson, D.E. (ed.), p. 37. Medical Examination Publishing Co: New York.

MACKELLAR, A., KEOGH, E.J. & LUGG, M. (1983). The undescended testis – 'lies, damned lies and statistics'. *Aust. Paediatr. J.*, **19**, 131 (Abstract).

MILES, B.J., KIESLING, V.J. & BELVILLE, W.D. (1985). Bilateral synchronous germ cell tumors. *J. Urol.*, **133**, 679.

MORRISON, A.S. (1976). Cyrptorchidism, hernia and cancer of the testis. *J. Natl Cancer Inst.*, **56**, 731.

MOSTOFI, F.K. (1973). Testicular tumors. Epidemiological, etiologic and pathologic features. *Cancer*, **32**, 1186.

NEWELL, G.R., MILLS, P.K. & JOHNSON, D.E. (1984). Epidemiologic comparison of cancer of the testis and Hodgkin's disease among young males. *Cancer*, **54**, 1117.

NEW SOUTH WALES CENTRAL CANCER REGISTRY (1981). *Cancer in New South Wales. Incidence and Mortality 1977*. Health Commission of New South Wales: Sydney.

PIKE, M.C., CHILVERS, C. & PECKHAM, M.J. (1986). Effect of age at orchidopexy on risk of testicular cancer. *Lancet*, **i**, 1246.

POTTERN, L.M., BROWN, L.M. & HOOVER, R.N. (1986). Cryptorchidism and testicular cancer. *J. Natl Cancer Inst.*, **77**, 832.

POTTERN, L.M., BROWN, L.M., HOOVER, R.N. & 4 others (1985). Testicular cancer risk among young men: role of cryptorchidism and inguinal hernia. *J. Natl Cancer Inst.*, **74**, 377.

PUGH, R.C.B. (1976). (ed.) *Pathology of the Testis*. Blackwell Scientific Publications: Oxford.

ROBSON, M.K., ANDERSON, J.M., GARSON, O.M., MATTHEWS, J.P. & SANDEMAN, T.F. (1981). Constitutive heterochromatin (C-banding) studies in patients with testicular malignancies. *Cancer Genet. Cytogenet.*, **4**, 319.

SCHOTTENFELD, D. & WARSHAUER, M.E. (1982). Testis. In *Cancer Epidemiology and Prevention*. Schottenfeld, D. & Fraumeni, J.F. (eds), p. 947. W.B. Saunders: Philadelphia.

SCHOTTENFELD, D., WARSHAUER, M.E., SHERLOCK, S., ZAUBER, A.G., LEDER, M. & PAYNE, R. (1980). The epidemiology of testicular cancer in young adults. *Am. J. Epidemiol.*, **112**, 232.

SCORER, C.G. & FARRINGTON, G.H. (1971). *Congenital Deformities of the Testis and Epididymis*. Butterworths: London.

SENTURIA, Y. (1987). The epidemiology of testicular cancer. *Br. J. Urol.*, **60**, 285.

SHAPIRO, S.R. & BODAI, B.I. (1978). Current concepts of the undescended testis. *Surg. Gynecol. Obstet.*, **147**, 617.

SPITZ, M.R., SIDER, J.G., POLLACK, E.S., LYNCH, H.K. & NEWELL, G.R. (1986). Incidence and descriptive features of testicular cancer among United States whites, blacks and Hispanics, 1973–1982. *Cancer*, **58**, 1785.

STONE, J.M., CRUICKSHANK, D.G., SANDEMAN, T.F. & MATTHEWS, J.P. (1991). Trebling of incidence of testicular cancer in Victoria, Australia 1950–1985. *Cancer* (in press).

SWERDLOW, A.J., HUTTLY, S.R.A. & SMITH, P.G. (1987). Testicular cancer and antecedent diseases. *Br. J. Cancer*, **55**, 97.

SWERDLOW, A.J., HUTTLY, S.R.A. & SMITH, P.G. (1988). Is the incidence of testis cancer related to trauma or temperature? *Br. J. Urol.*, **61**, 518.

WHITAKER, R.H. (1970). Management of the undescended testis. *Br. J. Hosp. Med.*, **4**, 25.

WORLD HEALTH ORGANIZATION (1967). *Manual of the International Statistical Classification of Diseases, Injuries and Cause of Death*, 8th revision. WHO: Geneva.